# Estimation of serum levels of mast cells-related chymase, histamine and diamine oxidase in oral submucous fibrosis- a preliminary report

**DOI:** 10.1186/s12903-025-05772-2

**Published:** 2025-03-17

**Authors:** Harshkant Gharote, Arati Panchbhai, Dipali Joshi

**Affiliations:** 1 Department of Oral Medicine and Radiology, Sharad Pawar Dental College and Hospital, Datta Meghe Institute of Higher Education and Research, Wardha, 442107 India; 2 Central Research Laboratory, Datta Meghe Institute of Higher Education and Research, Wardha, 442107 India

**Keywords:** Oral submucous fibrosis, Mast cells, Histamine, Chymase, Diamine oxidase

## Abstract

**background:**

Mast cell infiltration in oral submucous fibrosis (OSMF) has been drawn in to play a definitive role in initiation, progression, and symptom like burning sensation. Degranulation products of mast cells like tryptase and chymase have been studied through immunochemistry. The presence of mast cells in close to fibroblasts certainly makes them play a pivotal role in initiation of fibrogenesis in oral mucosa. As OSMF involves the oropharynx and esophagus along with the oral mucosa, the role of certain systemic factors might be considered in this spread apart from local factors. Present study was planned to identify the serum concentrations of various mast cell secretions like histamine and chymase using enzyme-linked immunosorbent assay (ELISA). Further, diamine oxidase (DAO), an enzyme that metabolizes histamine, was included to evaluate any correlation with serum histamine.

**Methods:**

Thirty-nine participants were equally divided into 3 groups: OSMF patients, areca chewers without OSMF, and healthy controls. Serum samples collected by drawing blood were estimated for serum histamine, chymase, and diamine oxidase using ELISA kits.

**Results:**

ELISA findings revealed significant differences in the serum values of histamine and chymase while concentration of serum DAO was not significant among the three groups. There was a positive correlation between histamine and DAO levels in all the groups.

**Conclusion:**

Mast cell-related bioactive molecules may render a systemic effect during initiation and progression of OSMF. DAO levels may rise linearly to metabolize histamine as a physiological phenomenon.

## Introduction

### Rationale

Oral submucous fibrosis (OSMF), a chronic inflammatory disorder of the oral cavity, affects the mucosa and connective tissue by atrophy and fibrosis respectively. Further, it involves the oropharynx and esophagus and possesses high chances of malignant transformation. A recently proposed definition of OSMF describes the entity as progressive, irreversible collagen metabolic disorders induced by areca nut, a known carcinogen. Various factors have been suggested to activate the disease process including areca nut chewing, genetic predisposition, and immunologic processes [1, 2]. Areca nut habit is considered as a primary etiological factor with the prevalence rate of OSMF among chewers ranging between 6.3% to 21.5% in India [3]. Moreover, the treatment of OSMF revolves around the improvement in the quality of patient’s life. Thus, targeting the alleviation of clinical symptoms using steroids, herbal preparations and nutritional supplements like vitamins is the primary objective. Nonetheless, there are no existing measures that guarantee the prevention of malignant transformation of this entity [4, 5].

Various proinflammatory cytokines, prostaglandins, and growth factors have been associated with the pathogenesis of OSMF. Immunohistochemical studies on mast cell expression in OSMF suggest their role in chronic inflammation and immune response [6–8]. In oral mucosa, mast cells are distributed especially around the microvascular bed, in close approximation to the basement membranes of endothelial cells and the nerves [9]. Literature reveals that histamine released by mast cells contribute to fibrosis by stimulating fibroblast proliferation while chymase induces angiogenesis [10, 11]. Studies have found that infiltration of mast cells has a vital role in the initiation and progression of OSMF. The role of mast cell related serene proteases, chymase, and tryptase, has been implicated in OSMF and may participate in its malignant transformation. Thus, mast cells can act as a dual-edged sword in OSMF by participating in juxtaepithelial inflammatory reaction in initial phases through immune response and activating angiogenesis via serene proteases in later stages of the disease [12–14].

Accordingly, it can be evident that mast cell-related secretions have a major role to play in various diseases processes including OSMF. Besides, involvement of the oropharynx and esophagus may show variation in levels of biochemicals released by mast cell with effects on involved tissue. Additionally, diamine oxidase, an enzyme involved in the histamine metabolism may show variation in the serum levels in context to the mast cell related serum histamine levels.

### Objectives

With the above background for the role of mast cells, the present study was proposed to estimate serum levels of histamine, chymase, and diamine oxidase in OSMF and compare with levels of healthy individuals with and without areca habit using enzyme-linked immunosorbent assay (ELISA).

## Method

### Study design

The present study was planned as a pilot project after receiving the institutional ethical clearance.

The STROBE statement (strengthening the reporting of observational studies in epidemiology) was used for the description of the case control study. The study design included three groups as OSMF individuals and, healthy controls with and without any areca or tobacco habits as participants for data collection. ELISA was performed for the estimation of serum chymase, histamine, and diamine oxidase.

### Setting

The present study was conducted at the Datta Meghe Institute of Higher Education and Research, Wardha, India for collection of data between 1st June to 31st July 2024 after approval from the Institutional Ethical Committee. the patients visiting the Department of Oral Medicine and Radiology in the Dental Hospital were recruited by random selection by briefing about the study protocol and their willingness to participate. The sample storage and ELISA tests were conducted in the Central Research Laboratory unit.

### Participants

Thirty-nine individuals participated after the written informed consent and were divided into three groups of 13 subjects as OSMF group (Group A), healthy individuals with areca habit (Group B), and healthy individuals without areca habit (Group C). Individuals with OSMF were selected following the functional staging classification of OSMF proposed by More CB et al. The classification of functional stages is given as: interincisal mouth opening—up to or greater than 35 mm (M1), interincisal mouth opening between 25 and 35 mm (M2), interincisal mouth opening between 15 and 25 mm (M3) and interincisal mouth opening less than 15 mm (M4) [1].

The inclusion and exclusion criteria were put forth for the groups as follows:

#### Individuals with OSMF

All the Group A individuals were selected based on the above clinical classification. OSMF individuals along with the clinical presentation of other premalignant disorders like lichen planus, leukoplakia or erythroplakia were excluded from the study. Furthers, the patients with history of previous medicinal treatment or currently under any medical treatment for OSMF were excluded from the group.

#### Individuals with areca habit without OSMF

Individuals with areca habit of more than one-year duration were included in Group B. detailed clinical examination oral mucosa for any early changes of OSMF or oral mucosal conditions like leukoplakia and lichen planus were performed. Individuals with the presence of such clinical findings were eliminated from the group.

#### Healthy individuals

The group C Individuals selected were without any deleterious habits of tobacco consumption in any form such as cigarette, bidi, and tobacco with lime. Clinical examination ensured that they have healthy oral mucosa. Any healthy individual having any kind of oral lesions were excluded from the group.

The history of any systemic diseases or any medications like antihistaminic agents that can affect mast cell related functions were the common exclusion criteria for the entire study population.

### Variable

The variables in the present study were serum levels of chymase, histamine and diamine oxidase. The collection of the serum samples was planned for estimation of these variables using ELISA. The possible potential cofounders in this study were the use of antihistaminic agents and routine herbal medications like curcumin by the participants although the in-depth drug history was recorded. The antihistaminic agents prevent histamine release by mast cell stabilization. These medications block the histamine H1 receptor and vasoconstriction in the tissue [15]. Further, studies show that herbal medications like Curcumin inhibits antigen-mediated activation of mast cells and suppress the degranulation and secretion of tumor necrosis factor-α (TNF-α) and interleukin-4 [16]. The presence of histamine intolerance among study groups could act as an effect modifier which was excluded by the subjective evaluation.

### Data sources and measurements

After the explanation of the procedure, the informed written consent was obtained from each participant. Aseptic precautions were taken to draw the blood samples and were stored in a refrigerator for clotting and separation of serum.

#### Samples and assays

Finally, blood samples were collected for all the 39 participants, and centrifuged for five minutes at 5000 rpm after clot formation to separate serum. All the serum samples were stored in vials at -20 degrees Celsius for the assay. The estimation of serum chymase, histamine, and diamine oxidase levels were performed using ELISA for all the serum samples. The standard ELISA kits for all the markers were used for assay manufactured by EC Bio Labs, New Delhi, India. All the samples and standards were processed as per the manufacturer’s instructions. The microplates were run in an ELISA reader set at an absorbance at 450 ± 10 nm.

### Bias

While no overt biases were observed in the study, the selection of OSMF patients and healthy individuals with an areca habit was based solely on a detailed clinical oral examination. This approach may overlook potential histological changes in the oral mucosa caused by harmful habits, presenting a possible source of bias. Likewise, patients with OSMF may display dysplastic alterations that remain undetectable without further investigation. However, a comprehensive clinical examination was conducted to identify early, visible signs of mucosal changes. Additionally, ethical constraints prevented the use of invasive procedures on the study population.

### Study size

The published statistics shows that the prevalence of OSMF in India is 7.21% [17], the sample size can be calculated using the formula for known population proportion (p). Further, as the study plan has unknown responses for factors to be estimated, sample size determination was done using the formula for phase II clinical trial [18]. The sample size selection will be at a 95 per cent confidence interval with margin of error (d) at ± 15%. Thus, the following formula can be used to calculate sample size:

$$n= \frac{{z}^{2 }p(1-p)}{{d}^{2}}$$

In the formula:

n = sample size.

z = z score (1.96).

p = population proportion (0.0721).

d = margin of error (0.15)

$$n= \frac{{\left(1.96\right)}^{2 }0.0721\left(1-0.0721\right)}{{0.15}^{2}}$$

n = 11.4

Thus, a minimum of 12 individuals could be enrolled in each group for the study. Nonetheless, the 48 welled ELISA microplate needs eight wells for standards sparing 40 wells, we decided to select 13 individuals in each group for a balanced study with a total of 39 subjects.

### Quantitative variable

The present study focused on measuring serum levels of chymase, histamine, and diamine oxidase—key biomarkers linked to immune and inflammatory responses. Using ELISA testing, we precisely quantified these serum values across all three groups, providing valuable insights into their potential role in the studied conditions.

### Statistical method

Data obtained from the ELISA testing of all the serum samples was tabulated in an Excel sheet. The statistical analysis was performed using Epi Info version 7.2.5.0 (A Database and Statistics Program for Public Health Professionals, CDC, Atlanta, GA, USA, 2011) and Jamovi project software [19, 20]. Descriptive statistics, including means, standard deviations, and medians, were calculated to summarize the data. To assess differences in age and serum levels of chymase, diamine oxidase, and histamine across the groups, an Analysis of Variance (ANOVA) was conducted. Post-hoc comparisons were made using the Bonferroni and Holm’s tests to pinpoint significant differences between groups. Pearson’s correlation analysis was employed to explore the relationship between serum histamine and diamine oxidase within each group. Additionally, Receiver Operating Characteristic (ROC) curve analysis was used to evaluate the area under the curve (AUC) and establish cutoff points for the biomolecular data across the three groups. A *p*-value of < 0.05 was considered statistically significant for all analyses.

## Results

### Participants and descriptive data

Thirty-nine individuals were divided into three equal groups, comprising of undistributed number of males and females due to random selection of study population and their demographic data including mean age and mouth opening is described in Table 1. All the participants were matched for age in years, the comparison of the age range was not significant statistically (*p* = 0.993). The mean opening of OSMF was significantly reduced as compared to Group B and Group C (*p* = 0.00001). The comparison of serum values for histamine and chymase among all the groups were statistically significant with *p* values as 0.0003 and 0.00001 respectively but DAO levels were statistically not significant (*p* = 0.06) (Table 2).

**Table 1:** Demographic data of the study population

Parameter	Males (N)	Females (N)	Total (N)	Age in years (Mean ± SD)	Mouth opening in mm (Mean ± SD)
Group A	10	3	13	32.61 ± 7.8	30.4 ± 9.31
Group B	7	6	13	32.23 ± 10.46	42.27 ± 2.02
Group C	9	4	13	32.3 ± 9.12	42.87 ± 2.01

**Table 2:** Comparison of mean age, serum histamine, chymase and DAO in all the groups

Parameter	Group A Mean ± SD	Group B mean ± SD	Group C mean ± SD	*p*- value
Age (Years)	32.61 ± 7.8	32.23 ± 10.46	32.3 ± 9.12	0.993
Mouth opening in mm	30.4 ± 9.31	42.27 ± 2.02	42.87 ± 2.01	0.00001
Serum Histamine (ng/ml)	0.97 ± 0.08	2.44 ± 1.68	7.65 ± 6.72	0.0003
Serum chymase (pg/ml)	93.59 ± 31.8	29.49 ± 6.07	34.37 ± 11.46	0.00001
Serum DAO (ng/ml)	0.27 ± 0.16	0.40 ± 0.31	0.19 ± 0.15	0.06

### Outcome data

The post-hoc Bonferroni test identified the pair-wise statistical relation between the groups. For serum histamine, pairwise comparison showed a significant difference in values between control versus chewers (*p* = 0.006) and control versus the OSMF groups (*p* = 0.0004). Whereas, serum levels of histamine were not significant between chewers and the OSMF group (*p* = 1.063) (Table 3). Serum chymase levels between controls and chewers were not significant (*p* = 1.6) but the comparison of OSMF group with chewers and controls was significant (*p* < 0.05) (Table 4). Further, there was no significant difference in serum DAO levels in pairwise comparison for all the groups (Table 5). There was a strong positive correlation between DAO and histamine levels in all the groups that was statistically highly significant (*p* = 0.00001) (Table 6).

**Table 3:** Pair-wise comparison of serum histamine levels

Groups	Bonferroni t-statistic	*p*-value	Inference
Group C vs. Group B	3.3219	0.006	Significant
Group C vs. Group A	4.2599	0.0004	Significant
Group B vs. Group A	0.9380	1.063	Insignificant

**Table 4:** Pair-wise comparison of serum chymase levels

Groups	Bonferroni t-statistic	*p*-value	Inference
Group C vs. Group B	0.6271	1.6	Insignificant
Group C vs. Group A	7.6147	< 0.05	Significant
Group B vs. Group A	8.2417	< 0.05	Significant

**Table 5:** Pair-wise comparison of serum DAO levels between the groups

Groups	Bonferroni t-statistic	*p*-value	Inference
Group C vs. Group B	2.4537	0.057	Insignificant
Group C vs. Group A	0.9383	1.06	Insignificant
Group B vs. Group A	1.5154	0.41	Insignificant

**Table 6:** Correlation of serum DAO with histamine levels between the groups

Parameter	Pearson’s R	*p*-value	Inference
Between Group C	0.9889	< 0.00001	Significant
Between Group B	0.9259		
Between Group A	0.9731		

**Fig. 1:**
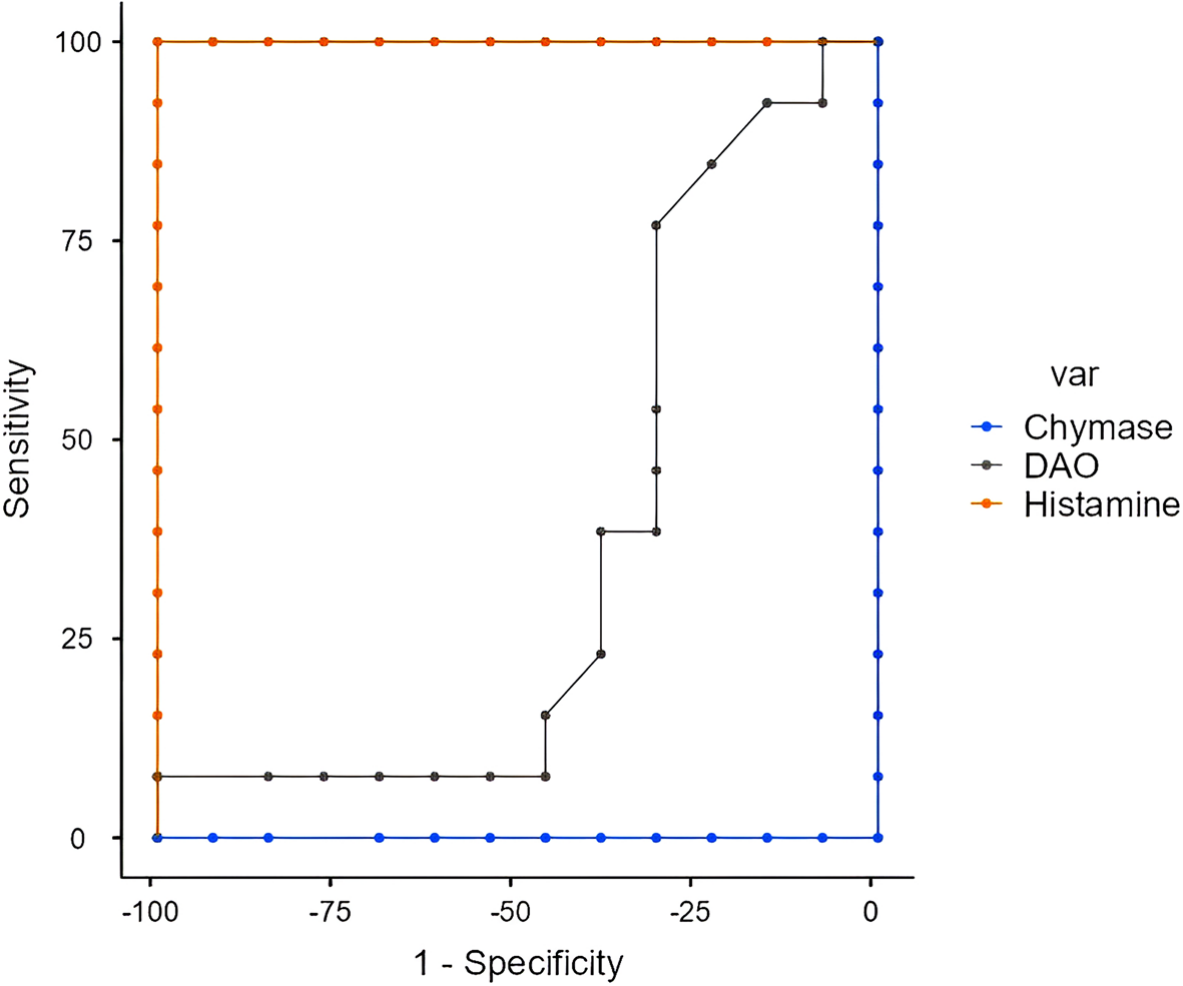
ROC curve for chymase, histamine and DAO between controls and OSMF

**Fig. 2:**
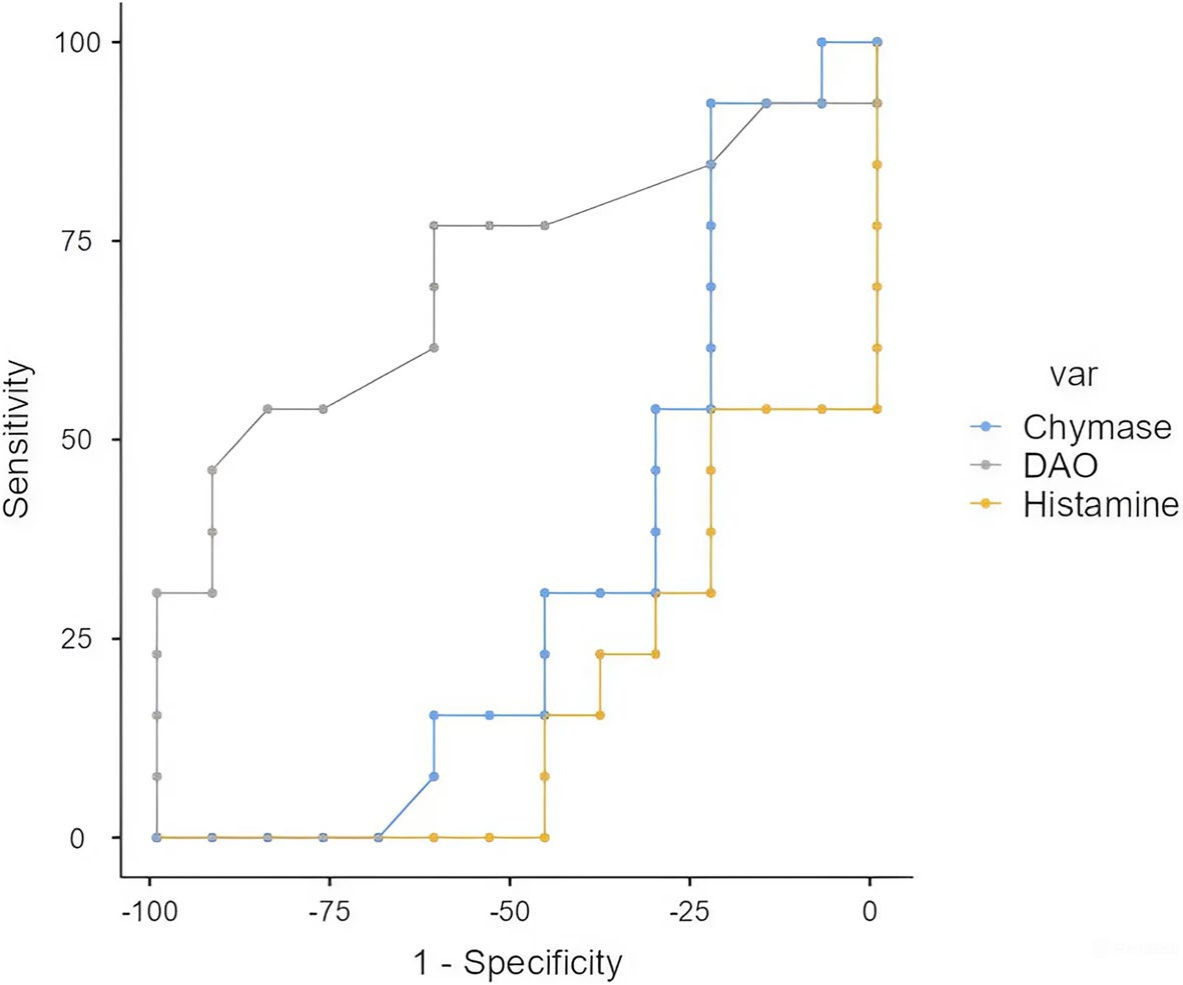
ROC curve for chymase, histamine and DAO between control and chewers

**Fig. 3:**
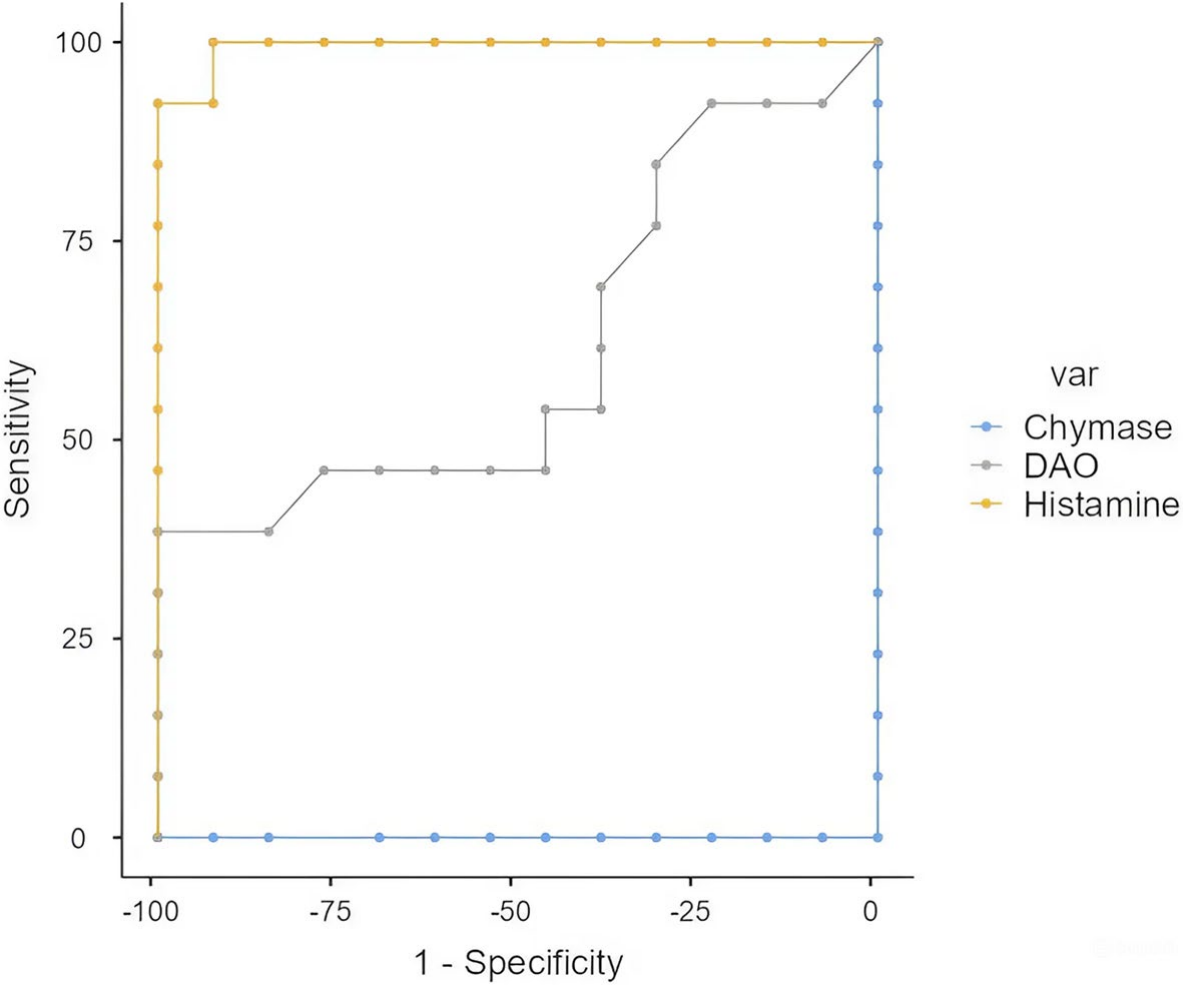
ROC curve for chymase, histamine and DAO between chewers and OSMF

The AUC was analyzed for histamine, DAO and chymase between the groups (Table 7, Figs. 1, 2 and 3). AUC values were determined by following the de Hond et al. labeling guidelines for the interpretation of area under the ROC curve [21]. The cutoff values for histamine between control-OSMF and chewer-OSMF groups were 1.58 and 1.12 ng/ml respectively with the excellent AUC suggesting the best possible serum level for histamine. Whereas, the control-chewer group exhibited failed expression of cutoff for histamine. In control-chewers and chewers-OSMF groups, DAO cutoff values were 0.259 and 0.556 with moderate AUC. The control-OSMF group had a random cutoff with a weak representation of AUC. Serum chymase showed poor expression of cutoff values among all the groups.

**Table 7:** ROC cutoff analysis for histamine, DAO and chymase between the groups

Molecule	Groups	Cutoff	Sensitivity	Specificity	PPV	NPV	AUC	AUC labeling
Histamine (ng/ml)	Control vs. Chewers	1.091	100%	0%	50%	NA	0.178	Poor
	Control vs. OSMF	1.58	100%	100%	100%	100%	1	Excellent
	Chewers vs. OSMF	1.12	92.31%	100%	100%	92.86%	0.994	Excellent
DAO (ng/ml)	Control vs. Chewers	0.259	53.85%	84.62%	77.88%	64.71	0.707	Moderate
	Control vs. OSMF	0.084	84.62%	23.08%	52.38%	60%	0.364	Poor
	Chewers vs. OSMF	0.556	38.46%	100%	100%	61.9%	0.615	Moderate
Chymase (pg/ml)	Control vs. Chewers	22.32	92.31%	23.08%	54.55%	75%	0.334	Poor
	Control vs. OSMF	14.19	100%	0%	50%	NA	0.00	Poor
	Chewers vs. OSMF	19.45	100%	0%	50%	NA	0.00	Poor

## Discussion

Mast cell hyperplasia is a common observation in fibrotic tissue and is documented in conditions like scar formation, post-traumatic joint fibrosis, burn fibrosis, and so on. Mast cells are normally residing within the mucosa and connective tissues of the oral cavity, skin, and gastrointestinal mucosa. Mast cell proteases may initiate alterations in basement membranes of oral mucosa during inflammation causing disruptions allowing entry of cytotoxic lymphocytes in the epithelium [9, 22]. In the connective tissue, their close juxtaposition to small venules and capillaries permits mast cell degranulation products to get released into local microcirculation. Mast cells are also found in intimate contact with fibroblasts and these cells can directly adhere to each other [22].

Likewise, in the pathological process of OSMF, mast cell infiltration has been studied over the last two decades and has been found to play a defined role in its initiation, progression, and development of burning sensation. Immunohistochemical studies have shown infiltration of mast cells and variation in their density was found with the progression of disease [2, 13]. Increased expression of mast cell tryptase and chymase in OSMF has been seen and found to associated with increased risk of malignant transformation [23].

The present study used ELISA for the estimation of chymase, histamine, and diamine oxidase levels in 39 individuals equally divided into OSMF group and healthy controls with and without areca habit. The comparison of mean age between all the groups was statistically significant.

Serum histamine levels were significantly lower in the OSMF group compared to the control groups, both with and without areca use. However, pairwise comparisons revealed no significant differences between the OSMF and chewer groups. The observed reduction in histamine levels could be linked to the properties of areca nut, known for its analgesic and anti-inflammatory effects through the inhibition of prostaglandins, leukotrienes, and histamine release [24]. Therefore, it can be inferred that individuals with areca use may exhibit decreased serum histamine levels, regardless of OSMF presence.

However, it is crucial to recognize that histamine has the potential to promote fibrogenesis and stimulate fibroblast proliferation. Previous studies have shown that histamine, when added to human lung fibroblast cell lines, boosts their proliferation. Additionally, experimental lung fibrosis research has demonstrated that mast cells interacting with alveolar macrophages trigger fibroblast proliferation, contributing to lung fibrosis [10, 25]. Moreover, Piera et al. conducted an animal study that highlighted histamine's role in directly increasing collagen content in heart myofibroblasts by enhancing procollagen type III expression [26]. In wound healing, histamine also stimulates the release of the profibrotic tumor growth factor-β1 (TGF-β1) from myofibroblasts within granulation tissue [27].

Further, a study on quantification of serum histamine in oral cancer showed non-significant levels within various histological grades but there was no control group for comparison [28]. Another study measuring serum histamine in various body cancers showed that the patients treated for malignancies showed normal levels of serum histamine as compared to significantly diminished levels during the cancer stage [29]. This implies that decreasing serum histamine in OSMF patients may help to identify evidence of malignant transformation.

The statistically significant increase in serum chymase levels in OSMF cases, compared to both chewers and controls, highlights its potential role in disease progression. In contrast, no significant differences were found between the chewer and control groups, suggesting that chymase may specifically contribute to fibroblast proliferation and subsequent fibrosis in OSMF. Veerappan et al. demonstrated that mast cell degranulation in pulmonary fibrosis releases renin, which acts on angiotensinogen to form Angiotensin I, later converted to Angiotensin II by chymase. This locally formed Angiotensin II can then stimulate fibroblasts, driving fibrosis [25]. A similar mechanism may be involved in the pathophysiology of OSMF. Furthermore, chymase playing essential role in initiating angiogenesis suggests that it could contribute to the malignant transformation of OSMF likewise, though the exact mechanism remains unclear.

Although the literature shows scarce data that evaluated serum/plasma chymase in potentially malignant disorders or malignancies, several studies have delineated its role in fibrosis and angiogenesis [10, 11, 25]. Chen et al. demonstrated role of chymase in promoting fibroblast proliferation and collagen synthesis by activating TGF-β pathway in hypertrophic scars [30]. This possibly explains the role of mast cell chymase in OSMF that is defined as a scarring disease with excessive collagen deposition as stated by Rao et al. [17].

Our findings reveal nonsignificant levels of serum DAO values among all the groups and also in pairwise evaluation. DAO was estimated to assess the relationship with histamine as metabolizes histamine by oxidative deamination. Nevertheless, it needs to be specified that DAO is a copper containing amine oxidase that exhibits relation with serum copper levels where in copper deficiency leads to reduced serum DAO activity [31]. However, on contrary to this relationship, OSMF patients show elevated serum copper levels due to high copper content in areca nut [32, 33]. Thus, the present study fails to support the exact mechanism for the diminished DAO levels in OSMF compared to healthy controls in view of high copper content in OSMF.

Correlation of serum histamine with DAO among all the three groups was significant with positive correlation. This indicates that the serum DAO levels are directly proportional to histamine that can be attributed to the role of DAO in histamine metabolism. Although the present study found a strong positive correlation between the two molecules, certain factors like food habits, irritable bowel disease, and non-celiac gluten sensitivity can act as confounding variables, while conducting the studies on a larger sample size these factors need to be emphasized. Further it would be interesting to note that low DAO levels are found in patients with histamine intolerance showing higher plasma histamine and a weak negative correlation. DAO supplementations in those patients have shown improvement in signs of histamine intolerance [34–36]. Therefore, diminished DAO levels in OSMF may suggest there is possibility of mechanism similar to histamine intolerance that mimics in OSMF.

The present study observed cutoff points of 1.58 and 1.12 ng/ml for serum histamine in controls and chewers against OSMF with excellent AUC inference. Therefore, it can be assumed that when any areca chewer exhibits serum histamine above 1.12 ng/ml may have higher chances of progression to OSMF. Moderate expression of cutoff point for DAO may be ascribed to its strong correlation with histamine metabolism. Thus, variations in serum DAO levels are only indicative and may not be diagnostic laboratory parameter in OSMF. This is in line to serum levels of DAO in histamine intolerance studied by Arih et al., where it is stated that these levels fail to discriminate between high probability and low probability histamine intolerance patients [34].

In present study, serum chymase levels in all the groups failed to show any reference point for cutoff. Poor AUC labeling for chymase indicates that this mast cell secretagogue has primary effect at the tissue level in OSMF and does not exhibit any systemic inference in the pathogenesis of OSMF in spite of higher serum levels compared to both the control groups. Similar inferences were made by Topparmak et al., wherein they found no role of chymase in systemic inflammation developed in obese hypertensive individuals [37].

However, there is a need to generate stronger evidence to establish any such facts through widespread research, as no published supporting literature particularly in context to OSMF is available in this regard.

### Limitations

This study represents a key advancement in understanding the role of mast cell-related bioactive molecules in the pathophysiology of OSMF. Although the small sample size limits the generalizability of the findings—representing only a fraction of the larger OSMF-affected population—the sample size is justifiable. Hertzog et al. recommend a minimum of 12 participants per group for pilot studies, while Whitehead et al. suggest a sample range of 10 to 40 participants, supporting the adequacy of the current sample for this exploratory research [38, 39].

### Interpretation

Comparing the various clinical grades of OSMF could reveal how each stage con tributes to the activation of fibroblasts and the progression of submucosal fibrogenesis. This approach has the potential to redefine OSMF as a disorder of collagen metabolism, opening avenues to investigate the involvement of mast cell-related products, such as interleukins, growth factors, and tryptase. Although the precise role of diamine oxidase remains unclear, targeted drug trials could explore its potential as a therapeutic inhibitor, halting OSMF progression at critical stages.

### Generalizability

Some studies have explained the role of chronic inflammation due to microtrauma to the oral epithelium that transpires the submucous fibrosis through proinflammatory cytokines, leukotrienes, and histamine [6, 24, 40]. Most of these chemicals are released by degranulation of the mast cells and play a pivotal role in the pathogenesis of OSMF. Thus, findings in this study can also be useful in establishing the role of inflammation in the pathophysiology of OSMF. Although serum chymase levels show significant increase in OSMF, it underscores the need to establish the further relationship with various stages of OSMF.

Burning sensation, a key clinical finding of OSMF, has been attributed to the mast cell infiltration in connective tissue wherein release of histamine is responsible for atrophy and itching of oral mucosa [2]. The diminished serum histamine levels in OSMF do not attribute to the symptom of burning sensation and suggest a localized mechanism of mast cell related histamine. Although we observed a linear relationship between DAO and histamine, there is a limited scope for DAO to contemplate in the pathogenesis of OSMF.

Further, the present study provides insight to future research in establishing the relationship between areca alkaloids, mast cells related chemicals and the immunohistochemical expression of these molecules in the affected oral mucosa. Likewise, expression of tissue histamine and histamine receptors to delineate their role in burning sensation, progression of fibrosis and malignant transformation needs to be established.

Thus, the present study endorses a need for robust study design with well-defined objectives and larger sample size to establish a relationship of locally infiltrated mast cells and their degranulation products in pathogenesis of OSMF.

## Conclusion

The key findings of this study highlight elevated chymase and reduced histamine levels in OSMF, underscoring the crucial role of mast cell secretions in fibroblast activation and subsequent fibrosis. DAO, an enzyme responsible for metabolizing histamine, shows a positive correlation with fibrosis progression, suggesting a regulated physiological relationship between these factors. However, to generate robust, impactful evidence, further studies are needed, focusing on both serum and tissue levels across larger, regionally diverse sample populations.

## Data Availability

The datasets used during the current study are available from the corresponding author on reasonable request.

## Abbreviations

OSMF: Oral submucous fibrosis
ELISA: Enzyme-linked immunosorbent assay
DAO: Diamine oxidase
TNF-α: Tumor necrosis factor-α
TGF-β1: Tumor growth factor-β1
